# Neurotoxicity and Underlying Mechanisms of Endogenous Neurotoxins

**DOI:** 10.3390/ijms222312805

**Published:** 2021-11-26

**Authors:** Yanlu Cao, Bo Li, Nafissa Ismail, Kevin Smith, Tianmei Li, Rongji Dai, Yulin Deng

**Affiliations:** 1 Beijing Key Laboratory for Separation and Analysis in Biomedicine and Pharmaceuticals, School of Life Science, Beijing Institute of Technology, Beijing 100081, China; 3120191351@bit.edu.cn (Y.C.); 3120191390@bit.edu.cn (T.L.); deng@bit.edu.cn (Y.D.); 2 Advanced Research Institute of Multidisciplinary Science, Beijing Institute of Technology, Beijing 100081, China; 3 Neuroimmunology, Stress and Endocrinology (NISE) Lab, School of Psychology, Faculty of Social Science, University of Ottawa, Ottawa, ON K1N 6N5, Canada; Nafissa.Ismail@uottawa.ca (N.I.); ksmit006@uottawa.ca (K.S.); 4 Brain and Mind Research Institute, University of Ottawa, Ottawa, ON K1N 6N5, Canada

**Keywords:** endogenous neurotoxins, neurotoxicity, neurodegenerative diseases, mechanism of action

## Abstract

Endogenous and exogenous neurotoxins are important factors leading to neurodegenerative diseases. In the 1980s, the discovery that 1-methyl-4-phenyl-1,2,3,6-tetrahydropyridine (MPTP) contributes to Parkinson’s disease (PD) symptoms led to new research investigations on neurotoxins. An abnormal metabolism of endogenous substances, such as condensation of bioamines with endogenous aldehydes, dopamine (DA) oxidation, and kynurenine pathway, can produce endogenous neurotoxins. Neurotoxins may damage the nervous system by inhibiting mitochondrial activity, increasing oxidative stress, increasing neuroinflammation, and up-regulating proteins related to cell death. This paper reviews the biological synthesis of various known endogenous neurotoxins and their toxic mechanisms.

## 1. Introduction

Neurodegenerative diseases include Alzheimer’s disease (AD), PD, Lewy body disease, amyotrophic lateral sclerosis (ALS), and Huntington’s disease (HD). It is estimated that the number of patients with neurodegenerative diseases will increase due to the aging of the global population. Neurodegenerative diseases are characterized by protein accumulation, synaptic reduction, neuronal dysfunction, and death [1]. Neurodegenerative diseases are not only affected by genetics, but also neurotoxins. Shaw’s group [2] proposed that neurotoxins could be an etiological factor for neurodegenerative diseases. For example, atypical Parkinsonism is greater in residents of Guadeloupe who consume a toxic compound containing “annonaceae”. Similarly, residents of Guam in the Western Pacific have a habit of eating flour that contains water-soluble neurotoxic factors, which results in high incidence of local ALS-Parkinsonism Dementia Complex (ALS-PDC). In AD, endogenous ammonia is a potential neurotoxin [3]. Neurotoxins have been shown to cause selective death of certain nerve cells and can be used to set up models of major neurodegenerative diseases such as PD, AD, and ALS [4]. Therefore, it is imperative to examine the role of neurotoxins in the development of neurodegenerative diseases.

Endogenous neurotoxins are produced by an abnormal metabolism of endogenous substances that affect the central nervous system (CNS). Some endogenous neurotoxins can cross the blood–brain barrier that delivers important oxygen and necessary nutrients to the brain [5]. Endogenous neurotoxins cause neurodegenerative diseases by affecting neuronal transmissions [6] that may be associated with blocking ion channels (e.g., K^+^, Ca^2+^, Na^+^) and receptors of ion channels of neurotransmitters (e.g., acetylcholine receptors), and inhibiting enzymatic activity (e.g., tyrosine hydroxylase (TH)) [7]. Most of the mechanisms of endogenous neurotoxins described in this review are related to inhibiting mitochondrial activity, increasing oxidative stress and neuroinflammation, and up-regulating apoptotic proteins [8]. The neurotoxicity of endogenous neurotoxins depends on its preferred accumulation sites in the brain, rate of synthesis, and tendency to form free radicals [9]. Yulin Deng’s group proposed a hypothesis on the mechanism of endogenous neurotoxins indicating that under oxidative stress, the body could trigger more aldehyde production. The aldehydes would react with endogenous amine, releasing more endogenous neurotoxins which would eventually lead to mitochondrial dysfunction and abnormal aggregation of α-Synuclein. This process would further exacerbate oxidative stress and mitochondrial damage in the progress of PD. This hypothesis links the relationship between oxidative stress, endogenous neurotoxins, and mitochondrial dysfunction systematically [10].

The effects of endogenous neurotoxins on PD have been widely studied in literature and are highlighted in this review. The main pathological symptom of PD is the degeneration of dopaminergic neurons in the substantia nigra pars compact (SNpc), resulting in the loss of dopamine in the striatum and the formation of Lewy bodies (α-Synuclein protein inclusions) [10,11]. α-Synuclein is a 14kDa protein widely expressed in the mammalian brain. α-Synuclein polymorphs (strains) are determined by both misfolded seeds and intracellular environments [12], and may influence the spreading of pathology and the cellular impact of the aggregates. The α-Synuclein/p25α strain induced by the multiple system atrophy-associated oligodendroglial protein p25α is highly prodegenerative in multiple system atrophy [13]. Poul Henning Jensen’s group [14] used organotypic hippocampal slice models and found that endogenous α-Synuclein aggregated in axons and cell bodies that spread to CA3 and CA1 regions in a prion-like manner, which was independent of phosphorylation at serine-129. Further, α-Synuclein aggregates also travel retrograde across synapses, along sensory nerves to pain processing areas in the central nervous system [15] or bidirectional via the vagus nerve, i.e., duodenum-to-brainstem-to-stomach [16]. α-Synuclein is not only the result of PD, but it is also a key factor of PD or neuronal death [17]. In PC12 cells, over-expressing α-Synuclein contributed to oxidative stress and increased endogenous neurotoxins. The increasement of endogenous neurotoxins aggravated mitochondrial dysfunction caused by α-Synuclein, suggesting that endogenous neurotoxins could link α-Synuclein with cell death [18]. It is speculated that α-Synuclein retrogradely propagates to the central nervous system, aggravating inflammation and oxidative stress, and promoting the formation of endogenous neurotoxins.

The development of new endogenous neurotoxins is hindered, probably because the process of neurodegenerative diseases is slow and complex. In addition, the limitation of human pathological samples increases the difficulty of research. This article reviews the mechanisms of action, neurotoxicity, and biological synthesis of endogenous neurotoxins, especially its relationship with PD.

## 2. Endogenous Neurotoxins

**Figure 1:**
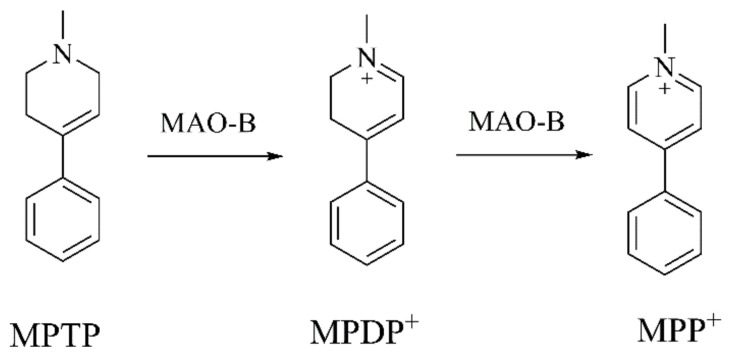
The metabolic process of MPTP [25].

Neurotoxin-induced neurodegenerative diseases originated in the 1980s following the discovery that MPTP, a byproduct of drug synthesis [19,20], induces PD symptoms, such as bradykinesia, postural instability, rigidity, cognitive deficits, and temporary autonomic disturbances [21,22]. Therefore, MPTP is commonly used to simulate the animal models of PD [23]. MPTP crosses the blood–brain barrier and oxidizes to 1-methyl-4-phenyl-2,3-dihydropyridinium (MPDP^+^) by monoamine oxidase B (MAO-B) and is converted into 1-methyl-4-phenylpyridinium (MPP^+^) [24,25] (Figure 1). MPP^+^ is transferred to dopaminergic neurons via dopamine transporter (DAT), and gathers in the mitochondria where it inhibits mitochondrial complex I. This process results in apoptosis by restraining electron transport chain, preventing oxidative phosphorylation and increasing reactive oxygen species (ROS), leading to mitochondrial dysfunction and dopaminergic neuron apoptosis. MPTP and MPP^+^ upregulate the expression of melastatin-like transient receptor potential melastatin 2 (TRPM2). This leads to Ca^2+^ entry and an increasing ROS level and caspase 3 activity, rendering cells more vulnerable to apoptosis [26]. Additionally, MPP^+^ activates 5′-adenosine monophosphate-activated protein kinase (AMPK), causing mitochondrial calcium uniporter deficiency and autophagic cell death [27].

Endogenous neurotoxins only attack one dopaminergic neuron at a time, which is consistent with the slow progression of neurodegenerative diseases [28]. Many findings show that endogenous neurotoxins play an important role in the pathogenesis of neurodegenerative diseases. There are three major sources of endogenous neurotoxins. First, MPTP structural analogues divide into tetrahydroisoquinolines (TIQs) and β-carbolines (βCs), following condensation of bioamines with endogenous small molecules. The second source is the quinones produced by dopamine oxidation. The third source is the metabolites of the kynurenine pathway.

### 2.1. Analogues of MPTP 

#### 2.1.1. Tetrahydroisoquinolines (TIQs)

**Figure 2:**
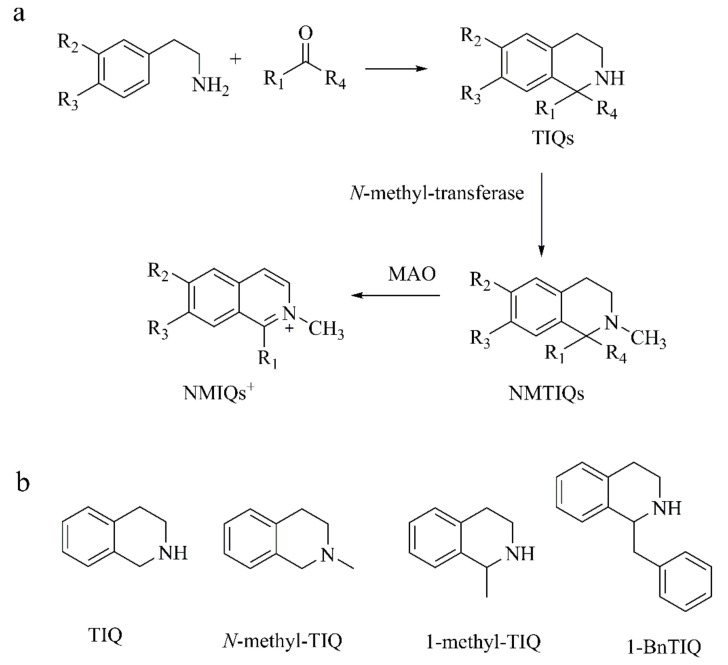
(**a**) The biosynthesis and metabolism of TIQs (R_1_: H, CH_3_, alkyl, aryl; R_2_: H, OH, OCH_3_; R_3_: H, OH, OCH_3_; R_4_: H, COOH) [31,32]; (**b**) the structure of TIQ, *N*-methyl-TIQ, 1-methyl-TIQ, 1-BnTIQ.

TIQs are widely distributed in plants, animals, and human brains, divided into catechol and non-catechol structures [29]. Most of them have been proposed as the potential pathogenic factors of PD. TIQs are formed by the condensation of bioamines with lipid peroxidation products or other biomolecular oxidative decomposition species, such as aldehydes or glyoxal [30,31]. TIQs are catalyzed by *N*-methyl-transferase to form *N*-methyl-tetrahydroisoquinolines (NMTIQs), and oxidized by monoamine oxidase (MAO) to obtain *N*-methylisoquinolium ions (NMIQs^+^) (Figure 2a) [32,33]. Using HPLC-EC and LC-MS/MS, M DeCuypere’s group [34] detected TIQs and its derivatives in all rodent and human brain regions, finding that the concentration of TIQ was higher than its methylated derivatives (1-methyl-1,2,3,4-tetrahydroisoquinoline (1-methyl-TIQ), *N*-methyl-TIQ) and benzylated derivative (1-benzyl-1,2,3,4-tetrahydroisoquinoline (1-BnTIQ)) in all brain regions (Figure 2b). TIQs induce damage to dopaminergic neurons through mechanisms similar to MPTP damage. We will discuss catechol tetrahydroisoquinolines such as 6,7-dihydroxy-1,2,3,4-tetrahydroisoquinoline (norsalsolinol), *N*-methyl-6,7-dihydroxy-1,2,3,4-tetrahydroisoquinoline (*N*-methyl-norsalsolinol), 1-methyl-6,7-dihydroxy-1,2,3,4-tetrahydroisoquinoline (salsolinol, Sal), 1(*R*),2(*N*)-dimethyl-6,7-dihydroxy-1,2,3,4-tetrahydroisoquinoline (*N*-methyl-salsolinol), 6,7-dihydroxy-1-(3′,4′-dihydroxybenzyl)-1,2,3,4-tetrahydroisoquinoline (tetrahydropapaveroline, THP), 1-acetyl-6,7-dihyroxy-1,2,3,4-tetrahydroisoquinoline (ADTIQ), and non-catechol tetrahydroisoquinoline, such as 1-BnTIQ and 1-methyl-TIQ, which is synthesized endogenously but has neuroprotective activity.

##### Norsalsolinol and N-methyl-norsalsolinol

**Figure 3:**
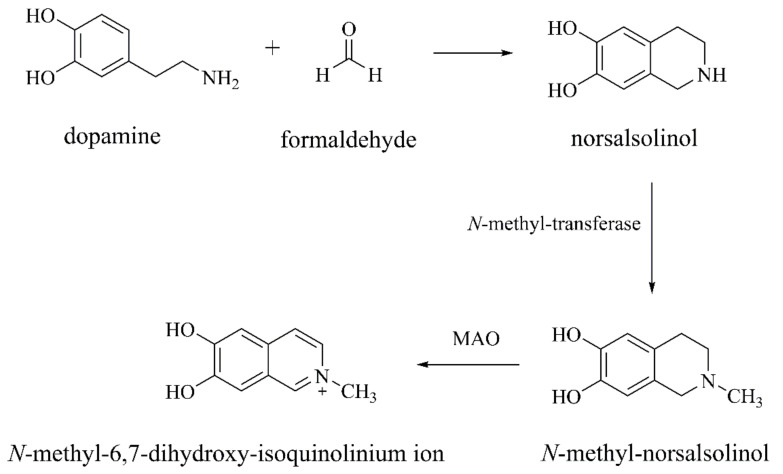
The biosynthesis and metabolism of norsalsolinol and *N*-methyl-norsalsolinol [32].

Norsalsolinol is formed by DA and formaldehyde through the Pictet–Spengler reaction (Figure 3) [35], which is selectively located in the substantia nigra (SN) and other dopamine-rich brain regions. Norsalsolinol damages SH-SY5Y cells (human neuroblastoma cells, a dopaminergic neuron cell model) by releasing cytochrome C, increasing ROS and inducing DNA oxidative damage and caspase-dependent apoptotic cell death [36]. Norsalsolinol can be transferred into *N*-methyl-norsalsolinol by *N*-methyl-transferase, which is oxidized by MAO to become *N*-methyl-6,7-dihydroxy-isoquinolinium ion (Figure 3) [32]. *N*-methyl-norsalsolinol is a weak neurotoxin that may be associated with the death of dopaminergic neurons in the nigrostriatal pathway [32] by inhibiting TH, an enzyme for DA synthesis [37].

##### Salsolinol and N-methyl-salsolinol

Salsolinol (Sal) is a strong neurotoxin which was first found in the urine of PD patients treated with L-DOPA in the 1970s [38]. Subsequently, it was also found in cerebrospinal fluid and in the brain tissue [39]. F. Musshoff’s group [40] found from twenty-five human samples who died of disease or violence that the ratio of (*R*)-Sal to (*S*)-Sal is about 2:1 in the human brain via solid-phase extraction procedure and gas chromatography-mass spectrometry (GC/MS). 

**Figure 4:**
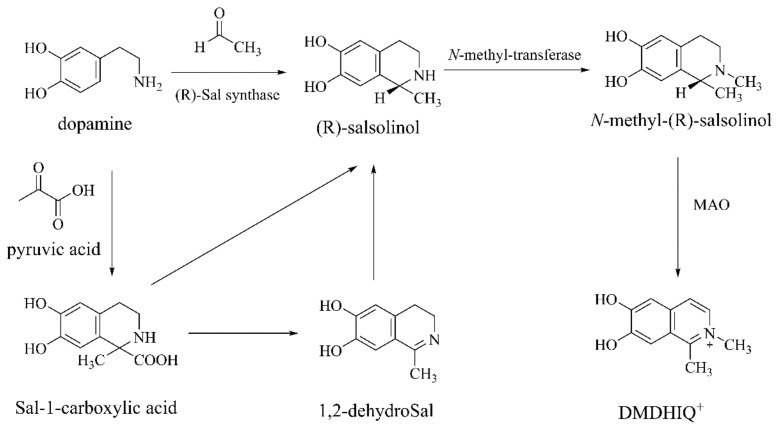
The biosynthesis and metabolism of (R)-salsolinol and *N*-methyl-(R)-salsolinol [45].

Sal contributes to alcohol addiction [41] and the production of neuroendocrine system-related hormones (e.g., prolactin [42], oxytocin [43]). Sal can be obtained from exogenous substances such as bananas, cheese, and alcoholic beverages. However, it is unclear whether exogenous Sal passes through the blood–brain barrier [44]. Sal may be synthesized by [45] DA and acetaldehyde through the Pictet–Spengler reaction to form racemic Sal. As well as, (*R*)-Sal can be achieved by the reaction of DA and acetaldehyde catalyzed by Sal synthase. DA and pyruvate can be catalyzed by Sal synthase to form Sal-1-carboxylic acid, which will then be decarboxylated and hydroreducted into Sal (Figure 4).

Sal inhibits the mitochondrial electron transport chain, inducing oxidative stress and mediating α-Synuclein to induce apoptosis. Villageliu and colleagues found that *Escherichia coli* produces Sal under an analogous intestinal environment containing DA, and adding alcohol raises the Sal content [46], suggesting that Sal might be generated by gut microbes, thus affecting neurological diseases. Although the neurotoxic effects of Sal have been extensively studied, the potential neuroprotective effects of low-dose Sal should not be overlooked. For example, previous findings show that [44] 10–250 μM of Sal produce neuroprotective effects rather than cytotoxicity and could reduce ROS and caspase activity caused by H_2_O_2_ or 6-OHDA. Furthermore, (*R*)-Sal can be converted into *N*-methyl-(*R*)-salsolinol by *N*-methyl-transferase. *N*-methyl-(*R*)-salsolinol is an endogenous neurotoxin found in urine and in the cerebrospinal fluid of PD patients and it is more toxic than Sal [47]. *N*-methyl-(*R*)-salsolinol can activate caspase-3 and increase oxidative stress, leading to the apoptosis of SH-SYSY cells [48]. *N*-methyl-(*R*)-salsolinol is oxidized into 1,2-dimethyl-6,7-dihydroxy-1,2,3,4-tetrahydroisoquinolineion (DMDHIQ^+^) by MAO [49] (Figure 4). Similar to MPP^+^, DMDHIQ^+^ induces apoptosis through the inhibition of mitochondrial complex I and the increase in ROS [50]. At present, Sal and *N*-methyl-salsolinol are used to construct cellular models of PD, rather than animal models.

##### THP

**Figure 5:**
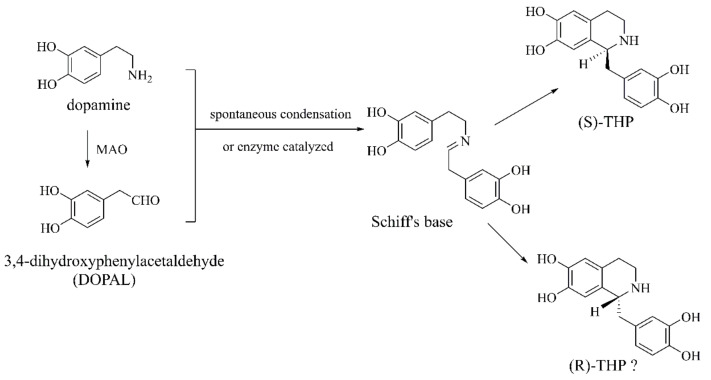
The biosynthesis of tetrahydropapaveroline (THP) (?-unknown) [52,54].

THP is also known as norlaudanosoline that is a precursor of endogenous morphine in mammals [51] and is detectable in plasma, urine, and in the brain of L-DOPA-treated PD patients [52] and can cross the blood–brain barrier [53]. THP is endogenously synthesized by DA and 3,4-dihydroxyphenylacetaldehyde (DOPAL, DA oxidative metabolite by MAO) (Figure 5) [52,54]. So far, only (*S*)-enantiomer has been detected in the human brain with the content at 0.12–0.22 pmol/g wet weight of brain tissue, indicating that THP synthesis is enzymatic [55]. THP is toxic to dopaminergic neurons not only in vitro but also in vivo [56]. THP does not cause lipid peroxidation, but instead promotes mitochondrial protein oxidation in the rat brain, and increases the content of protein carbonyl while decreasing protein-free thiol [52]. Furthermore, THP induces cell damaging through neurofilament-light (NF-L, a protein associated with neurodegenerative diseases) aggregation, generating carbonyl compounds [56]. Moreover, THP increases L-DOPA-induced apoptosis of PC12 cells by inhibiting mitochondrial complex I and α-ketoglutarate dehydrogenase [57].

##### ADTIQ

**Figure 6:**
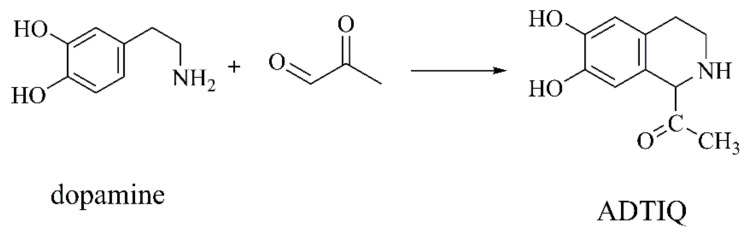
The biosynthesis of ADTIQ [58].

Abnormal glucose metabolism will produce a large amount of ROS that attack polyunsaturated fatty acids on the cell membrane, triggering lipid peroxidation, as well as activating aldehydes. Aldehydes react with DA to generate catechol tetrahydroisoquinolines through the Pictet–Spengler reaction. Yulin Deng’s group [17] first found the endogenous neurotoxin ADTIQ in the caudate nucleus, putamen, substantium, frontal cortex, and cerebellum of PD patients. ADTIQ, whose structure is similar to MPTP, is generated in vivo from DA and glycolytic intermediate methylglyoxal (Figure 6) [58]. ADTIQ activates the pro-apoptotic protein Bax, up-regulates caspase-3, reduces the anti-apoptotic protein Bcl-2, releases cytochromic C from mitochondria into cytoplasm, enhances cell oxidation, increases the expression of LC-3A, which is an mitochondria-dependent autophagy gene marker protein, and induces mitochondrial injury [59]. Methylglyoxal is associated with the development of diabetes [60], and ROS generation [61]. ADTIQ is a downstream product of the reaction between methylglyoxal and glucose in dopaminergic neurons, indicating that ADTIQ may be involved in causing dopaminergic neurotoxicity in hyperglycemia and diabetes. This provides a new view on the relationship between diabetes and PD [59].

##### 1-BnTIQ

**Figure 7:**
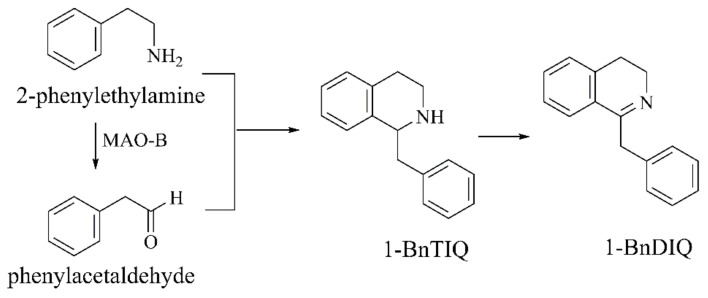
The biosynthesis and metabolism of 1-BnTIQ [62,65].

Non-catechol tetrahydroisoquinoline 1-BnTIQ, an endogenous neurotoxin, was found in the cerebrospinal fluid of PD patients. It is obtained by condensation of endogenous 2-phenylethylamine (PEA) and phenylacetaldehyde (a metabolite of PEA under MAO-B) (Figure 7) [62]. 1-BnTIQ can impair dopamine storage, contribute to the production of free radicals, and cause cell death [63]. 1-BnTIQ dose-dependently decreases the viability of SH-SY5Y cells and induces apoptosis, which is more pronounced than MPP^+^. 1-BnTIQ promotes lipid peroxidation, the expression of Bax, the formation of caspase-3, and reduces the expression of the anti-apoptotic protein Bcl-xL [62]. In 2013, Agnieszka Wasik’s group [64] demonstrated that 1-BnTIQ had neuroprotective activity at a low concentration of 50 µM or exhibited neurotoxic activity at a high concentration of 500 µM, inducing apoptosis of primary hippocampal cells, significantly enhancing glutamate-induced increasement in apoptosis markers. Yaichiro Kotake’s group [65] discovered that 1-BnTIQ could be metabolized to 1-benzyl-3,4-dihydroisoquinoline (1-BnDIQ) in rat liver S9 by CYP3A4 and CYP1A (Figure 7). 1-BnDIQ was more toxic and more lipophilic than 1-BnTIQ. 1-BnDIQ had stronger inhibition towards mitochondrial NADH-ubiquinone oxidoreductase and it might contribute to PD-related neurotoxicity.

##### 1-Methyl-TIQ

**Figure 8:**
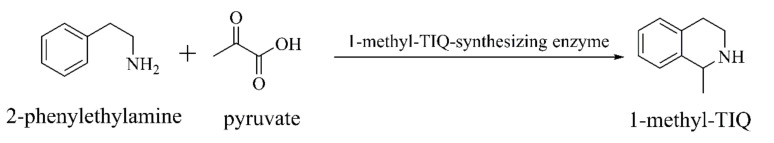
The biosynthesis and metabolism of 1-methyl-TIQ [67].

Some endogenous neurotoxins have protective effects at low concentrations, such as Sal and 1-BnTIQ. Quite differently, 1-methyl-TIQ only possesses neuroprotective property, according to the literature. 1-Methyl-TIQ is an endogenous amine synthesized in the brain of humans and animals. It can easily cross the blood-brain barrier. It can also be supplemented from foods such as bananas, cheese or red wine [66]. In the brain, it is obtained from 2-phenylethylamine (PEA) and pyruvate by 1-methyl-TIQ-synthesizing enzyme that is located in the mitochondrial-synaptosomal fraction (Figure 8) [67]. The neuroprotective mechanisms of 1-methyl-TIQ include inhibition of MAO, blocking MAO-dependent oxidation pathways; scavenging free radicals and inhibiting glutamate-induced excitatory toxicity, such as caspase-3 activity and lactate dehydrogenase concentration [68]. In addition, it can promote the synthesis of neurotrophic factors such as nerve growth factor (NGF), brain-derived growth factor (BDNF) and promote the growth and survival of neurons [69]. It is reported that TIQ also has similar neuroprotective effects and is also a reversible MAO inhibitor [70].

#### 2.1.2. β-Carbolines (βCs)

**Figure 9:**
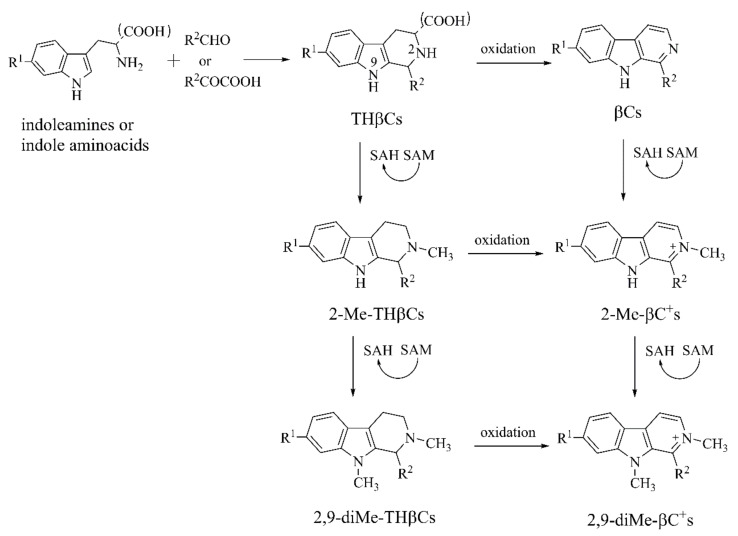
The biosynthesis and metabolism of βCs [31,73,75].

Other structural analogues of MPTP and MPP^+^ are βCs, which are naturally generated indoles alkaloids. They are harman alkaloids with a tricyclic pyrido[3,4-b]indole structure found in the plant *Peganum harmala* as well as *Passiflora incarnate* and *Bansteriopsis caapi*, which are used to treat asthma and jaundice [71]. They also exist in roast meat, coffee, corn, and soybeans [72]. In vivo, indoleamines fuse with aldehydes or α-ketoacids to form 1,2,3,4-tetrahydro-β-carbolines (THβCs), then oxidize into βCs (Figure 9) [31,73]. Central βCs accumulation may increase cell stress and apoptosis [31]. Neuromelanin, a pigment by-product of DA metabolism, combines with βCs to release dopaminergic neurotoxins [74]. βCs are further catalyzed by *N*-methyl-transferase (S-adenosyl-l-methionine (SAM)) to achieve *N*-methyl-βCs (Figure 9), potential endogenous neurotoxins related to PD [75]. We will discuss 9H-pyrido[3,4-*b*]indole (β-carboline, norharman), 1-methyl-9H-pyrido-[3,4-*b*]indole (1-methyl-β-carboline, harman), 2-methyl-β-carbolinium ion (2-Me-βC^+^), 2,9-dimethyl-β-carbolinium ion (2,9-diMe-βC^+^) and 1-trichloromethyl-1,2,3,4-tetrahydro-carboline (TaClo) in this section.

##### Norharman and Harman

**Figure 10:**
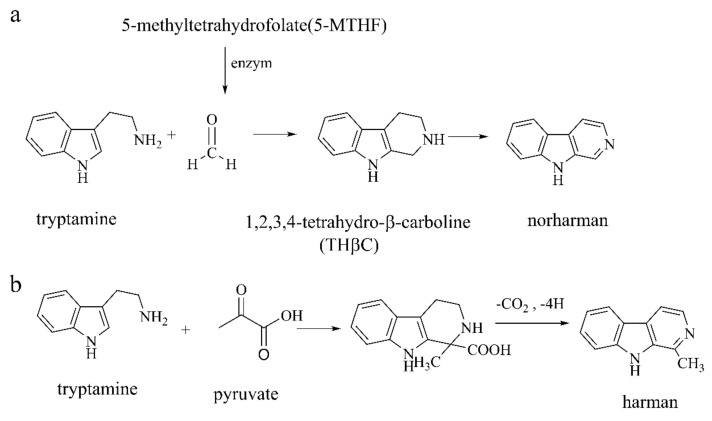
(**a**) The biosynthesis of norharman [77]; (**b**) the biosynthesis of harman [77].

Norharman and harman are present in the human brain and in the cerebrospinal fluid but increase 2–3 times in PD patients. Norharman is formed through the reaction of tryptamine with formaldehyde, the enzymatic product of 5-methylhydrofolate (5-MTHF) (Figure 10a) [76,77]. Norharman is obtained through endogenous condensation of tryptamine and pyruvate. It is an endogenous sedative that may stimulate specific β-carboline receptors and selectively inhibit MAO-A and B, acting like an antidepressant. Additionally, harman is obtained by endogenous condensation of tryptamine and pyruvate (Figure 10b) [76,77,78]. Harman can be generated either endogenously or exogenously through the diet (meat, roasted coffee beans, tobacco) [79], and exogenous acquisition is considered to be the main source [80]. Harman is associated with essential tremor (ET), but the mechanism is not clear [81]. Additionally, harman and norharman reduce the DA content in PC12 cells by down-regulating the activity of TH, thus increasing cytotoxicity [82]. Using *Caenorhabditis elegans* as the model, it is found that harman reduces mitochondrial activity and increases ROS in dopaminergic neurons, linking it to PD [79].

##### 2-Me-βC^+^ and 2,9-DiMe-βC^+^

In the brain, norharman is converted by SAM to 2-Me-βC^+^ and subsequently to 2,9-diMe-βC^+^ [83]. The content of 2,9-diMe-βC^+^ is 0.1 pmol/g tissue in the parietal association cortex and 0.77 pmol/g tissue in the substantia nigra [84]. 2,9-DiMe-βC^+^ mediated cell death by apoptosis and necrosis and it is more toxic than 2-Me-βC^+^ [85]. Specifically, 2,9-diMe-βC^+^ accumulates in the mitochondria and inhibits complex I of the respiratory chain, interferes with energy metabolism and reduces ATP content, and increases the free radicals and caspase-3.

##### TaClo

**Figure 11:**
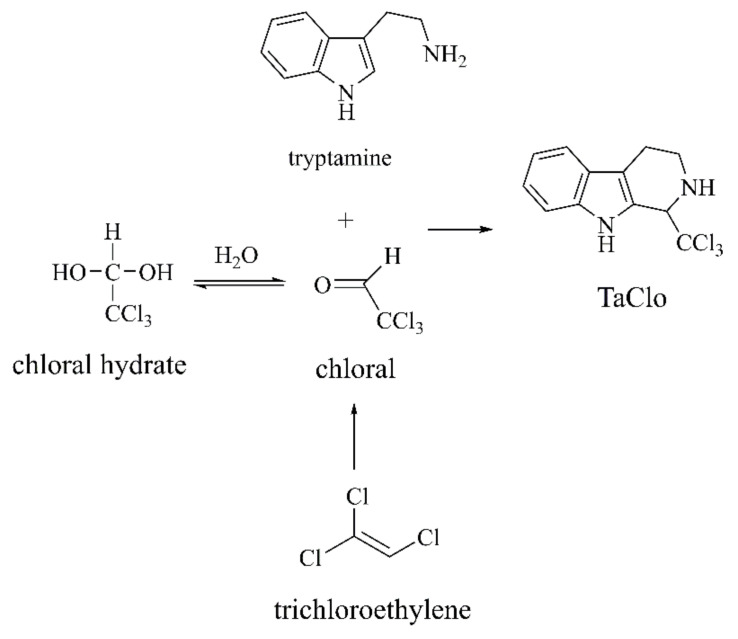
Condensation of tryptamine with chloral to form TaClo [86,87,88].

The environmental chemical trichloroethylene (TCE) is used as an industrial solvent. Interestingly, TCE can be inhaled and metabolized into chloral which reacts with endogenous tryptamine to generate endogenous neurotoxin TaClo, capable of crossing the blood–brain barrier (Figure 11) [86,87,88]. Sharma R.K.’s group [89] found that TaClo could accelerate the apoptosis of human neuroblastoma cell line SK-N-SH, inhibit mitochondrial complex I, increase the phosphorylation of AMPK, and inhibit the synthesis of neuronal prostaglandin E2 that is important for synaptic plasticity. Stereo injection of TaClo into the striatum of Wistar rats impairs mitochondrial complex I, increases ROS, and causes oxidative stress. In addition, TaClo elevates the expression of Iba-1, TNF-α, IL-6, Cox-2, and iNOS, which indicates microglial activation and an innate immune response [90]. Oral administration of TaClo can change the nocturnal spontaneous motor behavior of rats. Sontag T.A.’s group [91] proposed that TaClo may act as a pathogenic factor that crosses the mucosal barrier and induces neurodegenerative processes, ultimately affecting the entire brain.

### 2.2. Oxidation Products of Dopamine (DA)

DA is spontaneously oxidized into o-quinones and polymerized to form neuromelanin after being released into the cytoplasm. These o-quinones, such as dopamine-o-quinone (DA-o-Q) and aminochrome, are associated with neuromelanin-containing dopamine neuron damage [92]. DA is also oxidatively degraded by MAO, producing 3,4-dihydroxyphenylacetaldehyde (DOPAL), 3,4-dihydroxyphenylacetic acid (DOPAC), and homovanillic acid (HVA). The above oxidation processes produce endogenous neurotoxins [93] and hydrogen peroxide (H_2_O_2_) that creates the most toxic hydroxyl radical (⋅OH) [94], causing serious damage to nerve cells.

#### 2.2.1. Autoxidation Products of DA

**Figure 12:**
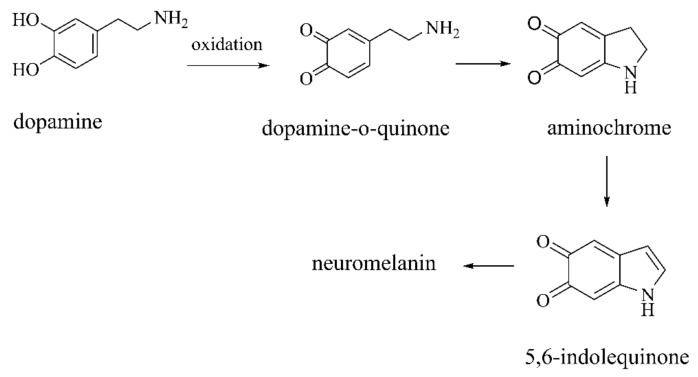
Dopamine spontaneously oxidization into neuromelanin [92,93].

Cytosolic DA is spontaneously oxidized by metals or enzymes [93], then polymerized into neuromelanin (Figure 12). However, dopaminergic neurons containing neuromelanin are prone to degeneration in PD patients [28,95]. This process relates to a series of o-quinone neurotoxins and neuromelanin produced during dopamine oxidation [92].

##### DA-o-Q

**Figure 13:**
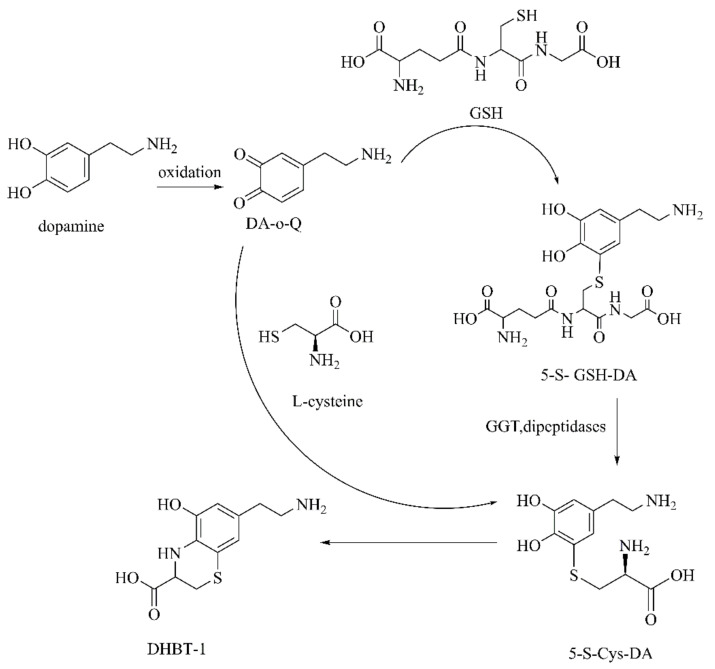
The reaction of DA-o-Q with biomolecules containing sulfur [97].

DA-o-Q lacks electrons, and reacts with central sulfur compounds to form highly toxic endogenous thio-catecholamines, which are highly toxic to dopamine neurons in the SNpc. DA-o-Q reacts with L-Cys to obtain three isomers dominated by 5-S-cysteinyl-dopamine (5-S-Cys-DA) [96], as well as reductive GSH, forming 5-S-glutathionyl-dopamine (5-S-GSH-DA). 5-S-GSH-DA is metabolized by gamma-glutamyl transferase (GGT) and dipeptidases into 5-S-Cys-DA. 5-S-Cys-DA is further oxidized to yield 7-(2-aminoethyl)-3,4-dihydro-5-hydroxy-2H-1,4-benzothiazine-3-carboxylic acid (DHBT-1) (Figure 13). The specific manifestations caused by these metabolites include mitochondrial dysfunction, ATP exhaustion, oxidative stress, protein carbonylation, DNA damage, and activation of apoptotic signals [97]. DA-o-Q can also adduct with proteins (e.g., parkin, DA transporter, TH, mitochondrial glutathione peroxidase 4, etc.). Among them, the modification of α-Synuclein contributes to stabilizing the toxicity of α-Synuclein protofibrils [93].

##### Aminochrome

Under physiological pH, DA-o-Q transforms into aminochrome by cyclization and oxidation. Aminochrome is the most stable o-quinone in the process of dopamine oxidation to neuromelanin [93]. Aminochrome can form adducts with proteins engaged in neurotoxic reactions [98], induce mitochondrial dysfunction, α-Synuclein aggregation, protein degradation dysfunction of lysosomes and proteasomes, neuroinflammation, and oxidative stress [28,95]. DT-diaphorase and glutathione S-transferase Mu-2 (GSTM2) prevent the neurotoxic effects of aminochrome in the body [28]. Aminochrome is a new inducer to set up a preclinical model of PD. The neurotoxicity of aminochrome takes place in the dopaminergic neurons, and the degenerative process is slow. These characters are more suitable for the pathological characteristics of PD [99]. Aminochrome oxidizes to form 5,6-indolequinone (Figure 12), the most reactive species under dopamine oxidation, which also forms neurotoxic protofibrils/oligomers with α-Synuclein [92].

#### 2.2.2. Oxidation Products of Dopamine by Monoamine Oxidase (MAO)

**Figure 14:**
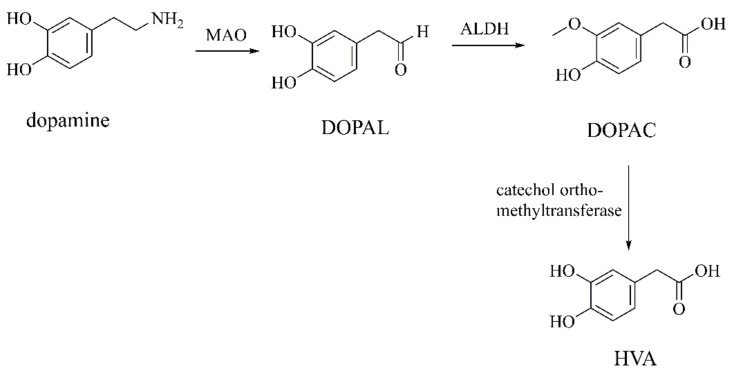
Dopamine oxidized by monoamine oxidase (MAO) [93,100].

In the central nervous system, MAO is present in the outer mitochondrial membrane of neurons, microglia, and astrocytes. MAO has two species: MAO-A and MAO-B. MAO-B can oxidize DA in human and MAO-A is applied in rats [100]. DA in the cytosol can be oxidized and degraded by MAO to produce toxic DOPAL. DOPAL is further oxidized to DOPAC by acetaldehyde dehydrogenase (ALDH), which is catalytically oxidized to HVA by catechol ortho-methyltransferase (Figure 14).

##### DOPAL

The aldehyde structure of DOPAL produces isoquinoline alkaloid tetrahydropapaveroline (THP) by reacting with DA. The pyrocatechol structure of DOPAL tends to be autoxidized to semiquinone radicals and o-quinones similar to DA, thereby producing ROS, aggravating oxidative stress of neurons, leading to DNA damage, protein cross-linking, and lipid peroxidation [101]. DOPAL can trigger the aggregation of α-Synuclein in vitro. α-Synuclein oligomers are neurotoxins that inhibit autophagy and proteasome activity and induce mitochondrial dysfunction, oxidative stress, as well as neuroinflammation [95]. Additionally, it is proposed that glyceraldehyde-3-phosphate dehydrogenase (GAPDH), an enzyme related to AD and PD, is a new target of DOPAL [102]. DOPAL also causes GAPDH aggregation and inhibits its activity, which may lead to detrimental Lewy body formation.

##### DOPAC and HVA

DOPAL can be further oxidized into DOPAC and HVA. DOPAC modulates nitric oxide (⋅NO) which induces cell death by inhibiting mitochondrial function and by not activating the caspase cascade [103]. HVA levels in the cerebrospinal fluid (CSF) decrease in dementia with Lewy bodies (DLB) and DLB with AD [104]. In addition, HVA combined with specific central proteins, such as t-tau, p-tau, and Aβ 1–42, is useful for distinguishing DLB, DLB with AD, and AD. However, HVA increases are associated with PD motor impairment [105]. This process is correlated with DOPAC concentration in the CSF, but not with DA concentration. Plasma HVA levels in HD patients also increase and are associated with the severity of the disease, but not with depression or dementia [106]. However, the mechanisms underlying the effect of HVA on neurodegenerative diseases require further investigation.

### 2.3. Metabolites of Kynurenine Pathway

**Figure 15:**
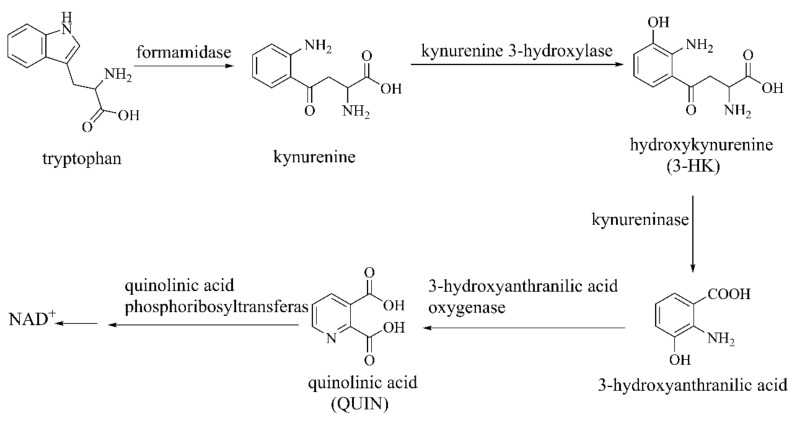
The biosynthesis of 3-HK, QUIN during kynurenine pathway [108,109].

The kynurenine pathway is the main metabolic pathway of the essential amino acid tryptophan, which exists in central glial cells and circulating inflammatory cells. The kynurenine pathway is regulated through redox and inflammatory components. This biometabolic pathway can generate neuroactive compounds such as hydroxykynurenine (3-HK) and quinolinic acid (QUIN/QA) [107]. Tryptophan is degraded to kynurenine by formamidase. Kynurenine is then metabolized into 3-HK by kynurenine 3-hydroxylase (kynurenine monooxygenase, KMO). 3-HK is then converted to 3-hydroxyanthranilic acid under kynureninase and oxidized to α-amino-ω-carboxymuconic acid semialdehyde, which can be rearranged nonenzymatically to form QUIN. Finally, QUIN is metabolized by quinolinic acid phosphoribosyltransferase, yielding nicotinic acid mononucleotide and the final product NAD^+^ (Figure 15) [108,109]. The metabolite levels are associated with aging and some neurodegenerative diseases [110]. KMO is an attractive neurological target for neurodegenerative and/or neuroinflammatory diseases, especially PD, AD, and HD. Peripheral inhibition of KMO has central neuroprotective effects [111]. 

#### 2.3.1. 3-HK

Central 3-HK increases in HD, dementia associated with human immunodeficiency virus (HIV) infection, hepatic encephalopathy, and PD, causing severe neurological dysfunction. Specifically, 3-HK is at nanomolar concentrations in CNS under normal circumstances; however, the level can increase up to three times in HD [110,112]. 3-HK in the brain may be derived from peripheral tissues through the blood–brain barrier and de novo synthesis in the brain, probably by astrocytes and microglia [113]. The nerve injuries caused by 3-HK may be mediated by free radicals rather than glutamate receptors [114]. An amount of 1–100 µM of 3-HK induces toxic effects on primary neurons extracted from the rat striatum, which could produce oxidative stress on neuron cells at pathological concentrations, resulting in the accumulation of peroxides, eventually leading to cell death [113].

#### 2.3.2. QUIN/QA

QUIN/QA is produced by dendritic cells. The concentration of QUIN is 50–1000 nM in the mammalian brain [108]. It is greater in HD, AD, ALS, and PD patients, and has neurotoxic, gliotoxic, and pro-inflammatory effects [115]. Its neurotoxicity is higher than 3-HK. The acknowledged mechanisms of QUIN-mediated cytotoxicity have been studied [116]. QUIN is a selective agonist of N-methyl-D-aspartate (NMDA) receptor. It overstimulates the NMDA receptor, increases the level of intracellular calcium giving rise to activate nitric oxide synthase (NOS), phosphoolipases, and protein kinases. It also increases ROS/RNS (reactive nitrogen species) [115]. In addition, QUIN enhances the release of synaptosomal glutamate, which inhibits glutamate entry into the astrocytes, resulting in accumulation of glutamate in the synaptic cleft and neurotoxicity. QUIN also suppresses antioxidant enzymes, promotes the production of ROS, and accelerates lipid peroxidation. QUIN inhibits mitochondrial complexes, leading to energy deficiency and apoptosis. Recently, it was proposed that QUIN accelerates amyloid oligomerization of 1N4R Tau in vitro, which is associated with AD [116]. This is another adverse factor of QUIN as an endogenous neurotoxin. In non-invasive surgery, administering QUIN that is unable to break through the blood–brain barrier normally can be directed to the specific locations of the brain, reducing the frequency of seizures [117].

## 3. Conclusions and Future Perspectives

It is widely believed that endogenous neurotoxins are important causes of neurodegenerative diseases. In conclusion, TIQs, βCs, and autoxidation products of DA are mostly hypothesized to contribute to PD, while oxidation products of dopamine by MAO are mostly hypothesized to be associated with DLB, HD, and AD. The metabolites of kynurenine pathway will increase in HD, PD, AD, and ALS. The progression of neurodegenerative diseases is long and complex, which makes it more difficult to find key and novel endogenous neurotoxins. At present, the general toxic studied mechanisms of neurotoxins include inhibition of mitochondrial function, reduction of ATP content, enhancement of oxidative stress causing ROS, destruction of protein function, causing neuroinflammation, and apoptosis. Neuroprotective therapies are mostly suggested to target mitochondrial dysfunction and neuroinflammation [118].

Future research could be aimed at further study of the mechanisms of known endogenous neurotoxins, refine the targets, discover other potential mechanisms to explore the relationship with neurodegenerative disease; and explore whether relatively small doses of endogenous neurotoxins provide protective effects. Endogenous neurotoxins and their metabolites can act as markers for the early diagnosis of PD. If the damaging cycle is broken by blocking the production and metabolism of endogenous neurotoxins, it may be possible to prevent the pathogenesis of PD [17]. The production of endogenous neurotoxins is mostly enzyme-catalyzed. It might be possible to use effective enzyme inhibitors to block the generation of endogenous neurotoxin. Special attention is suggested to be paid to α-Synuclein aggregates spreading from neuron to neuron, which may promote the production of endogenous neurotoxins, causing double damaging effects. In addition, it is worth to explore appropriate endogenous neurotoxins inducing neurodegenerative disease models that can not only show clinical symptoms, but also be consistent with the progressive nature of the diseases. This would help us conduct pathological studies to design new diagnostic biomarkers and to evaluate the potential of new drugs, new controlled release systems, new targeted drug delivery systems, and new therapies for rescuing the neurons and treating neurodegenerative diseases.

## Data Availability

Not applicable.

## Abbreviations

MPTP	1-methyl-4-phenyl-1,2,3,6-tetrahydropyridine
PD	Parkinson’s disease
DA	dopamine
AD	Alzheimer’s disease
ALS	amyotrophic lateral sclerosis
HD	Huntington’s disease
ALS-PDC	ALS-Parkinsonism Dementia Complex
CNS	central nervous system
TH	tyrosine hydroxylase
SNpc	substantia nigra pars compact
MAO	monoamine oxidase
DAT	dopamine transporter
ROS	reactive oxygen species
TRPM2	transient receptor potential melastatin 2
TIQs	tetrahydroisoquinolines
βCs	β-carbolines
norsalsolinol	6,7-dihydroxy-1,2,3,4-tetrahydroisoquinoline
*N*-methyl-norsalsolinol	*N*-methyl-6,7-dihydroxy-1,2,3,4-tetrahydroisoquinoline
Sal	salsolinol
THP	tetrahydropapaveroline
ADTIQ	1-acetyl-6,7-dihyroxy-1,2,3,4-tetrahydroisoquinoline
SN	substantia nigra
DOPAL	3,4-dihydroxyphenylacetaldehyde
NGF	nerve growth factor
BDNF	brain-derived growth factor
SAM	S-adenosyl-L-methionine
norharman	9H-pyrido[3,4-*b*]indole
harman	1-methyl-9H-pyrido-[3,4-*b*]indole
2-Me-βC^+^	2-methyl-β-carbolinium ion
2,9-diMe-βC^+^	2,9-dimethyl-β-carbolinium ion
TaClo	1-trichloromethyl-1,2,3,4-tetrahydro-carboline
5-MTHF	5-methylhydrofolate
ET	essential tremor
TCE	trichloroethylene
DA-o-Q	dopamine-o-quinone
DOPAL	3,4-dihydroxyphenylacetaldehyde
DOPAC	3,4-dihydroxyphenylacetic acid
HVA	homovanillic acid
3-HK	hydroxykynurenine
QUIN	quinolinic acid
NMDA	N-methyl-D-aspartate
NOS	nitric oxide synthase
AMPK	5′-adenosine monophosphate-activated protein kinase
